# PEG@ Carbon Nanotubes Composite as an Effective Nanocarrier of Ixazomib for Myeloma Cancer Therapy

**DOI:** 10.1186/s11671-022-03707-2

**Published:** 2022-08-05

**Authors:** Hanady A. Elgamal, Samah Abdelsabour Mohamed, Ahmed A. Farghali, Abeer M. E. Hassan

**Affiliations:** 1 Material Science and Nanotechnology Department, Faculty of Postgraduate Studies for Advanced Sciences (PSAS), Beni-Suef University, Beni-Suef, 62511 Egypt; 2 National Organization of Drug Control and Research, Dokki, Egypt; 3 Analytical Chemistry Department, Faculty of Pharmacy, October 6 University, Giza, Egypt

**Keywords:** Multi-walled carbon nanotubes, Anticancer drug, Cytotoxicity, RPMI 8226 cell line, Ixazomib, MLN2238C, PEGylation, Nanotechnology

## Abstract

In this work, the preparation of a PEG@ multi-walled carbon nanotubes (MWCNTs) composite has shown a great potential effect in tumor therapy using graphite powder at room temperature. PEGylated MWCNTs were created and used as a carrier for targeting the antineoplastic drug Ixazomib to myeloma cancer cells (abnormal plasma cells). Ixazomib (MLN2238) was covalently encapsulated into functionalized carbon nanotubes modified with polyethylene glycol (PEG 600) to obtain MWCNTs-PEG-MLN2238. The Ixazomib@ MWCNTs-PEG composite shows promising results as an effective nanocarrier and using a small amount of MWCNTs-PEG-Ixazomib that has a low toxicity compared with that of Ixazomib alone. A multifunctional MWCNTs-PEG-Ixazomib composite is used to test biological effects on multiple myeloma cell lines RPMI 8226 using the MTT assay to enhance treatment efficiency. The cytotoxicity of free Ixazomib citrate (69% cell viability of RPMI8226 cells) was higher than that of MWCNTs-PEG-Ixazomib (91% cell viability) at the same maximum concentration of Ixazomib citrate (50 µg/ml). In this work, we performed a study of preparation of MWCNTs with an acceptable Ixazomib loading efficiency and determination of the drug systemic toxicity for the first time. In this study, the preparation of MWCNTs with acceptable Ixazomib loading efficiency and determination of the drug systemic toxicity was performed for the first time. The MTT assay results show decreasing the toxicity of Ixazomib after loading with the MWCNTs-PEG composite. The MWCNTs-PEG @ Ixazomib show promising results as an effective carrier of Ixazomib and lead to a decrease in the cost of using Ixazomib.

**Graphical Abstract:**

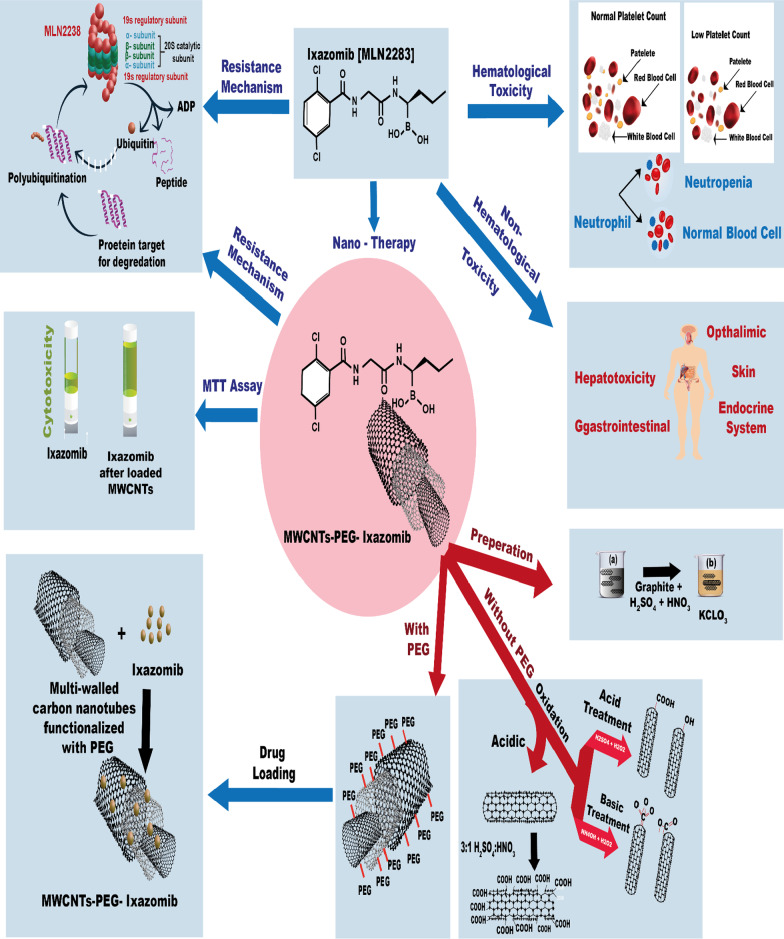

**Supplementary Information:**

The online version contains supplementary material available at 10.1186/s11671-022-03707-2.

## Introduction

Cancer considered is one of the top three diseases that cause death in modern society. Multiple myeloma (MM) is a cancer that forms in bone marrow cancer and often spreads in other areas of the body, such as the skull, ribs, spine, and pelvis. There are many treatments for multiple myeloma such as immunomodulatory drugs, anthracyclines, and proteasome and histone deacetylase inhibitors [1]. Ixazomib is an antineoplastic chemotherapy drug classified as a proteasome inhibitor and is known as Ninlaro (MLN2238). Ixazomib is considered the first proteasome inhibitor (PI) oral drug for patients affected by multiple myeloma and was approved by the FDA in November 2015. The biological activity of Ixazomib is released when it contacts the gastrointestinal tract and plasma due to hydrolyzed Ixazomib components to free boric acid metabolites in aqueous solutions [2]. The most common side effects of the use of Ixazomib are hematological toxicities such as thrombocytopenia [3] and Neutropenia [4] non-hematological toxicity, which includes gastrointestinal toxicity, hepatotoxicity, nervous system toxicity and ophthalmic toxicity [5, 6]. Side effects increase with increasing the amount of drug intake [7]. Consequently, different nanomaterials have been suggested to decrease side effects of the drug during its administration.

Nowadays, carbon nanotubes, nanofibers, halloysite tubes and other nanomaterials that have an effective pathway to enter the medical market and use in various biomedical applications, show unique properties (physical, chemical, or structural) such as high surface area, high aspect ratio, effective therapeutic agent loading, attractive nanocarriers, smart nanoprobe and other unique properties [8, 9]. Carbon nanotubes are needle-shaped structures with a high length-to-diameter ratio and it is advantageous to pass through cell membranes [10]. Moreover, their large surface area and inner hollow space are an excellent scaffold for the attaching of drug molecules [11]. The stability of MWCNTs allows various chemical modifications to their tips and surfaces, improving their biocompatibility and binding to the bioactive agents as well [12, 13]. In the last decade, different studies have been performed and developed to add multifunctional properties to MWCNTs, such as generating covalently bound functional groups for multi-walled carbon nanotubes using strong acids (H_2_SO_4_ and HNO_3_) for nanomedicine applications [13, 14]. Applications of MWCNTs have increased rapidly due to their oxygen-containing moieties on the surface that can be modified by inorganic or organic molecules to affect the enhancement of the solubility of MWCNTs [15, 16]. Basic Functionalization of MWCNTs has been proved that it gives better dispersion stability, which made it selected for drug loading as the best method. MWCNTs can be functionalized through two pathways, non-covalent and covalent pathway that is functionalized carbon nanotubes by adding a new attachment by creating a covalent bond between both two skeletons. The covalent pathway to functionalize carbon nanotubes can be divided depending on attached with (C(=O)OH) groups on the surface of multiwalled carbon nanotubes into two subpathways, direct covalent sidewall, and indirect covalent [17, 18]. The replacement of hydroxyl in C(=O)OH) groups with chloride followed by a reaction to form esters or amides has a significant impact on increasing the reactivity of MWCNTs [19, 20]. MWCNTs modified with PEG, a bifunctional spacer, show potential therapeutic applications to extend blood circulation time, be compatible with living tissues and apply as a carrier for antitumor therapeutics as [21]. Hence, PEGylated MWCNTs have been used as delivery systems for various of anticancer agents including cytostatic [22, 23], or antisense oligonucleotides [24]. Furthermore, PEGylated MWCNTs can be used as a unique multi-functionalization composite [25] to identify and therapy potential human diseases (theranostics applications).

The current study aims to prepare a PEGylated MWCNTs composite as a promising nanocarrier for Ixazomib. To our knowledge, no study was done on Ixazomib citrate conjugated to MWCNTs -PEG as an effective nanocarrier of oral anticancer drug which can be less toxic than the free drug. In this research, the MWCNTs-PEG 600 composite was prepared, Ixazomib citrate was encapsulated into the PEGylated MWCNTs, and PEGylated MWCNTs-Ixazomib composite was investigated by different effective techniques such as FTIR, DSC, SEM, TGA, TEM, and DTG. Drug loading, cytotoxicity, and encapsulation efficiency were investigated. We highlighted the effectiveness of PEGylated MWCNTs-Ixazomib composite for treatment of multiple myeloma cancer cells and its superior anticancer activity over native Ixazomib citrate.

## Materials and Methods

### Materials

Ixazomib was received as a gift from Takeda Pharmaceuticals U.S.A., Inc. MWCNTs with  > 98% carbon basis, O.D. × L 6–13 nm × 2.5–20 μm, size 10 μm length, and 12 nm outer diameter, (CAS no: 308068-56-6) were purchased from Sigma Aldrich and Graphite powder (< 20 μm, 99.995 + % purity, 45Im, B. No:282863 Aldrich). Polyethylene glycol (Mw = 6000 g mol^−1^) Acid nitric (HNO_3_), tetrahydrofuran (THF), dimethylformamide (DMF), triethylamine (TEA), sodium hydroxide (NaOH), hydrochloric acid (HCl), sulfuric acid (H_2_SO_4_), oxalyl chloride (COCl_2_) B.NO: 08801 was purchased from Sigma-Aldrich and received from Acros Organics Co., Ltd. Methanol, acetonitrile, potassium chlorate, and all other chemicals and reagents were also purchased from Sigma-Aldrich (St. Louis, MO, USA).

### Instrumentation

#### Preparations Instruments

The general preparation and functionalization of MWCNTs were performed under Vacuum lab line ductless fume hood (FH-250). Ultrapure deionized water was obtained from Milli-Q® Integral for Water Purification (Milli-Q® Advantage A10 Ultrapure Water Purification System). All samples were weighed using calibrated RADWAG electronic balance (XA60/220/X). The ultrasonication process was performed by FALC Ultrasonic Bath to (> 20 kHz), Model: LBS2-10 to agitate particles in the samples and to produce a good dispersion of samples in solutions. Centrifugation was performed using Hettich-type EBA 12. MWCNTs samples were filtered by a Deschem 1000 ml system that is connected with a pump, which was classified under a W/Vacuum type to accelerate the filtration process, after that, all samples were dried in an ECO CELL drying oven with natural air convection (50/60 Hz).

#### Characterization Instruments

To determine the drug loading and the content of Ixazomib was quantified by HPLC (Agilent 1260 Infinity with autosampler). General characterization and techniques were carried out by Fourier Transform Infrared Spectra (Shimadzu FT-IR Prestige-21) model using KBr tablets Nicolet Nexus 870 to examine the chemical bonding and functional groups of the samples and to study the drug loading, all samples were mixed with potassium bromide with a 1–2:200 ratio. Then, the mixture was pressed into a round cake and characterized in the scanning range of 400–3500 cm^−1^. To determine the actual drug loading, TGA was performed on a NETZSCH STD 409 C/CD Instrument, at CMRDI laboratory, Temp.Cal/Sens.files:Tcalzero.tcx, Model of meas:DSC-TG/Sample, Atmosphere: He/50/…,Crucible: DSC/TG Pan Al_2_O_3_ Thermogravimetric/Differential Thermal Analyzer with a heating rate of 10 °C/ min from room temperature to 900 °C in He. To determine the Ixazomib content, the different weights of MWCNTs-PEG-Ixazomib between 50 and 800 °C were calculated. Field emission scanning electron microscopes (QUANTA -FEG250, Japan) are used to characterize the morphology of MWCNTs after and before acidification and MWCNTs-PEG-Ixazomib. Transmission electron microscope (JEOL-JEM 2100) TEM operated at 200 keV to observe the internal structure of samples. The cytotoxicity of MWCNTs-PEG-Ixazomib and Ixazomib to RPMI 8226 cell line was assessed by the MTT assay. The final step of the MTT assay was performed using a Perkin-Element UV spectrometer.

### Preparation of Carbon Nanotubes from Graphite Powder

First, 2.0 g graphite (99.995% purity, 45Im, Sigma Aldrich) was gradually added to 100 mℓ of fuming acid solution (HNO_3_:H_2_SO_4_ with amount 1:3) for 60 min followed by a process of cooling the mixture in an ice bath at 5 °C and the obtained solution was stirred for 60 min after 10.0 g of potassium chlorate was added gradually and carefully during this process, so a lot of heat was released. The next step is heating the obtained solution after adding potassium chlorate up to 120 °C overnight (for 11 h) and then putting the heating solution of graphite that includes potassium chlorate in the air for 72 h to obtain unreacted graphite precipitated on the bottom and floating reacted MWCNTs that stirred for 60 min after adding it to 1000 ml of DI water, followed by immediate filtration and drying of the final mixture. The steps from adding the solution to DI water to drying were repeated 6 times to obtain high-quality MWCNTs with low impurities.

#### Sample Characterization

TEM was investigated using JEOL instrument (model JEM 2100 and operated at 200 keV) and used to identify the morphology of MWCNTs as shown in (Additional file 1: Fig. S1s)., MWCNTs-PEG-Ixazomib and other samples.

### Functionalization of Carbon Nanotubes

**Fig. 1:**
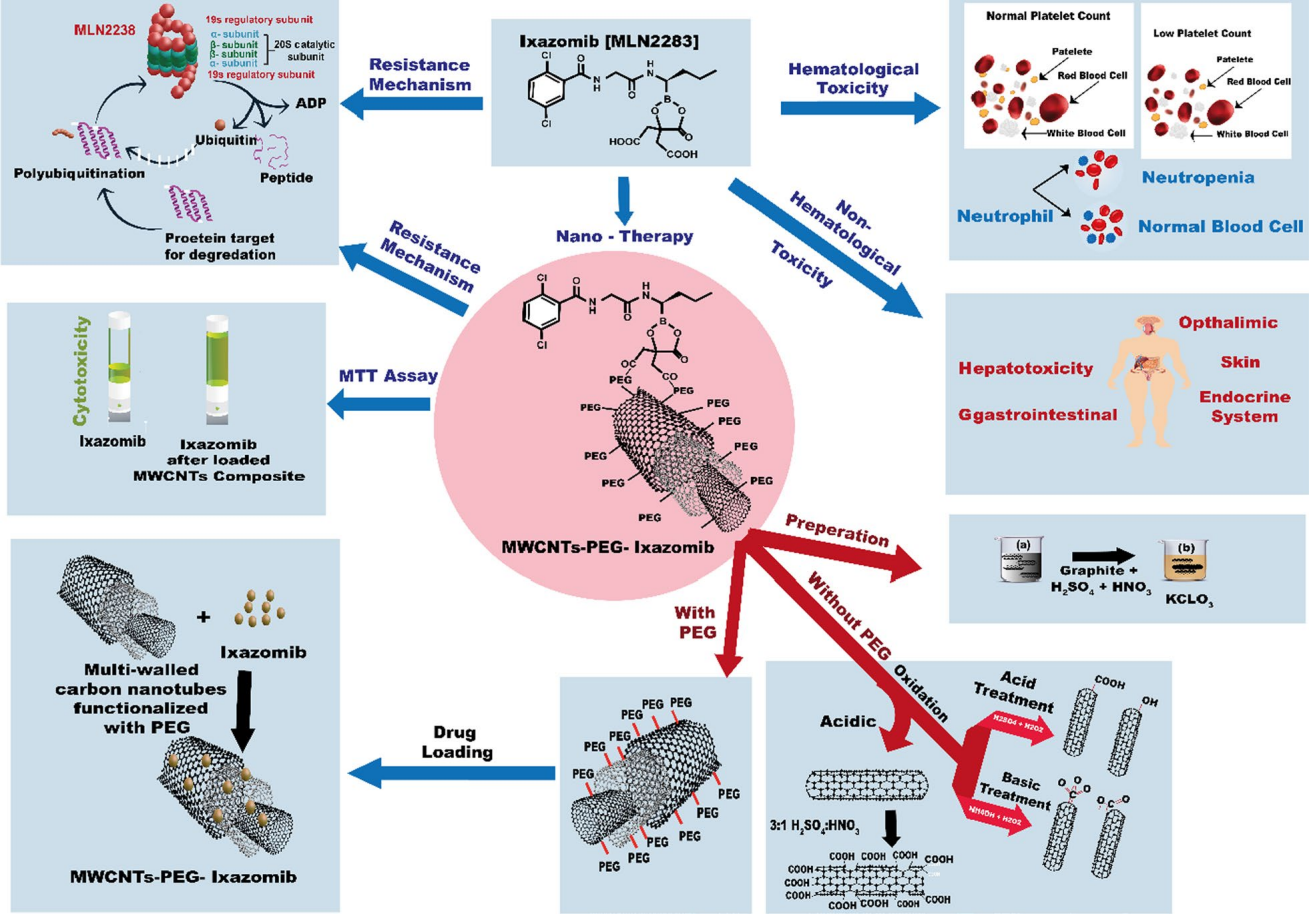
**a** the structure of Ixazomib loaded multi-walled carbon nanotubes. **b** Schematic illustration of Hematological toxicity of Ixazomib on normal platelet count and neutrophil. **c** Schematic illustration of non-hematological toxicity of Ixazomib on human organs. **d** Method of preparation of multi-walled carbon nanotubes. **e** Mechanism of multi-walled carbon nanotubes without polyethylene glycol (PEG). **f** Mechanism of multi-walled carbon nanotubes with polyethylene glycol (PEG). **g** Schematic illustration of loading Ixazomib on multi-walled carbon nanotubes. **h** Cytotoxicity of Ixazomib before and after loaded MWCNT (I) Mechanism resistance of Ixazomib

As shown in the graphical abstract (Fig. 1), MWCNTs treatments can be categorized into different pathways depending on the covalent functionalization [25].

- The first pathway is to modify the surface of MWCNTs with acidic treatment

This pathway is to be used to add covalent functionalization properties to the surface of MWCNTs and produce carboxylated MWCNTs by adding the MWCNTs to a mixture of HNO_3_ and H_2_SO_4_, followed by adding the obtained solution to hydrochloric acid. In this method, 1 g of MWNCTs (Sigma Aldrich) was placed in a 500 ml round bottom flask and 50 ml of a mixture of H_2_SO_4_: HNO_3_ in a ratio of 3:1 was added to form an acidified MWCNTs mixture that was stirred for 15 min. Then, the mixture was refluxed at temperature 65–70 °C for 30 h, then the mixture was diluted with 250 ml of highly pure deionized water, filtered using 0.45 µm PTFE Millipore, washed with ultrapure water until the pH = 7 after each wash filtration is conducted and the resulting precipitate after reaching pH = 7 was suspended in a mixture of (30%) H_2_O_2_: H_2_SO_4_ (4:1 v/v) then refluxed of the mixture was carried out for 2 h at 70 °C. To improve the purity of MWCNTs and remove metal catalysts and unwanted materials, 50 ml conc HCl was added to the resulting suspension and sonication was conducted for 30 min and filtration with a PTFE Millipore filter. The resulting mixture was dried under vacuum at 120 °C overnight.

(b) The second pathway is to produce oxidized MWCNTs

This pathway is to be used to add covalent functionalization properties to the surface of MWCNTs and produce oxidized MWCNTs by using NH_4_OH and H_2_O_2_ and then adding HCl. 0.5 gm of MWCNTs was added to a mixed solution between NH_4_OH (25%) and H_2_O_2_ (30%) with the same volume to volume ratio, then the mixture was kept for 5 after heating up to 800 °C in a condensation system. After that, the obtained dispersion from the condensation system was diluted in water and filtered with a PTFE Millipore filter under vacuum. To obtain a dried and pure composite, the produced material was dried at 600 °C under vacuum after washing several times with ultrapure water to adjust PH meter results to obtain neutral pH.

### Selection of the Optimized Pathway for MWCNTs Functionalization

Depending on the dispersion stability, the selection of the optimized pathway for MWCNTs functionalization was made by dispersing 10 mg of functionalized MWCNTs into 10 ml PBS solution (pH 7.4). The obtained solution was sonicated for 10 min and kept in sealed vials to compare the dispersion stability of the fresh mixture and after 15 days, as shown in Additional file 1: Fig. S2s a, b.

### Preparation of PEGylated Multiwalled Carbon Nanotubes (MWCNTs-PEG)

100 mg of functionalized MWCNTs was taken in a conical flask and subsequently 20 mL of 68% oxalyl chloride, the mixture was stirred for 6 h. Oxalyl chloride was evaporated under vacuum. The dried powder was dispersed in 20 mL of anhydrous DMF. 1 gm PEG 600 was added to the dispersed mixture to form the PEG 600-dispersed mixture by stirring for 120 h at 100 °C. After flirtation through a 0.2 mm PTEF membrane filter, the mixture was rinsed with DMF, ethanol and deionized water. After using a centrifuge under the following conditions 25,000 rpm for 600 s at 20 °C, the MWCNTs were washed with deionized water, the previous step was repeated 7 times to form a natural solution. After that, 2 mL of deionized MilliQ water was used to adjust the volume. The MWCNTs-PEG preparation process was started by heating mixing 10 mg of MWCNTs and 1 mL of crude PEG solution at 25 °C for 20 min to form MWCNTs-PEG solution containing unbound PEG600, which removed by repeating the centrifugation process at the same conditions with discarding the supernatant and dissolving the residue (MWCNTs-PEG) in 1 mL of H_2_O and storing the solution at 40 °C to use it for further applications. To test their solubility in water, a certain amount of MWCNTs with or without functionalization were dispersed in water of a specific volume under 30 min of ultrasonication, and the dispersion of MWCNTs was then centrifuged at 1000 rpm for 5 min, if there was no precipitation following the centrifugation, MWCNTs were considered soluble at such concentration, i.e., the solubility in water.

### Modification of MWCNTs-PEG with Ixazomib Citrate

Ixazomib incorporation into MWCNTs-PEG (Encapsulation of Ixazomib into MWCNTs):

Ixazomib was incorporated into the inner cavity of MWCNTs using the nano-extraction method [26]. The MWCNTs- PEG composite (50 mg) was dispersed in 10 ml of acetonitrile in a 50 ml volumetric flask by sonication for 60 s then 20 ml of 20 mM CBS (citrate buffer solution) was added then Ixazomib (100 mcg) was added to the suspension and sonicated again for 30–60 s to completely dissolve. The volume was diluted and stirred at room temperature for 3 days to facilitate the attachment of Ixazomib to the surface of MWCNTs-PEG. After stirring MWCNTs-PEG-Ixazomib for 3 days, the centrifugation process at 5000 rpm for 30 min was started to leave MWCNTs-PEG-Ixazomib at the bottom, while the retained supernatant was used for further characterizations and identifications. The mixture still contained an excessively unreacted amount of Ixazomib which was rinsed with acetonitrile and deionized water to eliminate the unincorporated drug. After 3 cycles of washing and centrifugation, the black residue was filtered through a 0.2 mm filter membrane to obtain purified MWCNTs-PEG-Ixazomib that was stored at 5 °C in dry space after the freeze-drying process was applied to use in other tests and applications and the amount of unreacted Ixazomib was calculated using HPLC. HPLC analysis of Ixazomib citrate was performed with a Luna C8 column (4.6 mm id × 150 mm ID, grain size 3 μm). The mobile phase (A):0.01% TFA in water (v/v) and mobile phase (B):0.01% TFA in acetonitrile (v/v). Both mobile phases were used in gradient mode. The injection volume and the flow rate were 10 μl and1.0 ml/min, respectively. The column temperature and sample tray were optimized at 25 °C and 5 °C, respectively. The UV detector wavelength was optimized at 220 nm.

The loading capacity of Ixazomib Citrate in MWCNTs-COOH was determined using this equation:

1 $${\text{Drug}}\;{\text{loading}}\% = (W{\text{total}} - W{\text{free}})/W{\text{total}} \times { 1}00\%$$

*W*free is the analyzed weight of free Ixazomib in the supernatant.

*W*total is the analyzed weight of Ixazomib used in the formulation.

2 $${\text{Encapsulation}}\;{\text{efficacy}}\;{ }\left( {{\text{EE \% }}} \right) = \left( {\frac{{{\text{Amount }}\;{\text{of}}\;{\text{ Ixazomib}}\;{\text{ in}}\;{\text{ the}}\;{\text{ nanoparticles }}}}{{{\text{Amount}}\;{\text{ of}}\;{\text{ Ixazomib}}\;{\text{ initially}}\;{\text{ added }}}}} \right) \times 100$$

All data were calculated using an average of three measurements (Additional file 1: Fig. S3s a & b).

The obtained powders were observed using Fourier Transform Infra-Red (FTIR), Scanning Electron Microscopy (SEM) and transmission electron microscopy (TEM), also TGA, DSC and DTG were measured.

### Cell Culture and MTT Assay

The multiple myeloma RPMI8226 Cell Line cells were obtained from the American Type Culture Collection; cells were cultured using DMEM (Invitrogen/Life Technologies) supplemented with 10% FBS (Hyclone), 10 ug/ml insulin (Sigma), and 1% penicillin–streptomycin. All other chemicals and reagents were purchased from Sigma or Invitrogen. Plate cells (cells density 1.2–1.8 × 10,000 cells/well) in a volume of 100 µl complete growth medium + 100 ul of the tested compound per well in a 96-well plate for 24 h before the MTT assay. Rinse the cell layer with 0.25% (w/v) Trypsin 0.53 mM EDTA solution. Add 2.0 to 3.0 ml of Trypsin EDTA solution to the flask and observe cells under an inverted microscope until the cell layer is dispersed usually within 5 to 15 min. Add 6.0 to 8.0 mL of complete growth medium and aspirate cells by gently pipetting. Centrifuge approximately 125×g for 5 to 10 min. Discard the supernatant. Resuspend the cell pellet in a fresh growth medium. Add appropriate aliquots of the cell suspension to new culture vessels. Incubate cultures at 37 °C for 24 h. After the treatment of cells with serial concentrations of the compound to be tested, incubation is carried out for 48 h at 37 °C, then the plates were examined under an inverted microscope and proceed with the MTT assay.

#### MTT Cytotoxicity Assay Protocol

The MTT method for monitoring in vitro cytotoxicity is well suited for use with multiwall plates. For the best results, cells in the log phase of growth should be employed and the final cell number should not exceed 106 cells/cm^2^. Each test should include a blank containing a complete medium without cells. Incubate culture for 2–4 h depending on cell type and the maximum cell density. After the incubation period, dissolve the resulting culture and formazan crystals by adding an amount of MTT solubilization solution [M-8910] equal to the volume of the original culture medium. Spectrophotometrically measured absorbance at a wavelength of 450 nm. Tests performed on multiwall plates can be read using the appropriate type of plate reader or the contents of individual wells may be transferred to appropriate size cuvettes for spectrophotometric measurement.

#### MTT Cell Viability Assay

The RPMI8226 human multiple myeloma cell lines were obtained from the American Type Culture Collection, U.S.A. The cytotoxicity of Ixazomib Citrate, MWCNTs, MWCNTs-PEG, and MWCNTs-PEG-Ixazomib were first evaluated with a MTT method. To compare the efficacy of the drug-loaded (MWCNTs-PEG-MLN2238) against free drug Ixazomib, twelve concentrations (between 0.1 and 100 μm) of free Ixazomib were initially chosen for the MTT assay [27, 28] involving RPMI8226 cells. The experiment was repeated three times. The percentage of cell survival was calculated as in Eq. (3): [29]

3 $${\text{Surviving}}\; {\text{fraction}} = \frac{{{\text{O}}.{\text{D}}. \left( {{\text{treated}}\;{\text{cells}}} \right)}}{{{\text{O}}.{\text{D}}. \left( {{\text{control}}\;{\text{cells}}} \right)}}$$

## Results and Discussion

The chemistry of MWCNTs has been developed to introduce various functional groups on the surface and extremities of MWCNTs with the purpose of changing its physicochemical properties and binding to drug molecules [12]. Various hydrophilic molecules were attached to MWCNTs to facilitate their suspendability in water or organic solvents and to decrease their toxicity, which is a great consideration in the development of MWCNTs as a vehicle for drug delivery. Therefore, in this study, the following techniques have been performed according to the next steps to obtain MWCNTs functionalized with PEG to improve their hydrophilicity and solubility of different MWCNTs in water, which increased their degree of functionalization.

### Preparation and Functionalization of oMWCNTs

The best pathway for functionalization of MWCNTs is by refluxing MWCNTs in concentrated HNO_3_ and H_2_SO_4_ acids to generate anchor moieties that can be modified to add other functional groups as a subsequent PEGylation to form MWCNTs-PEG.

### Characterization of the Pristine, Functionalized MWCNTS, Drug, MWCNTs-PEG-Drug

#### FTIR Analysis

**Fig. 2:**
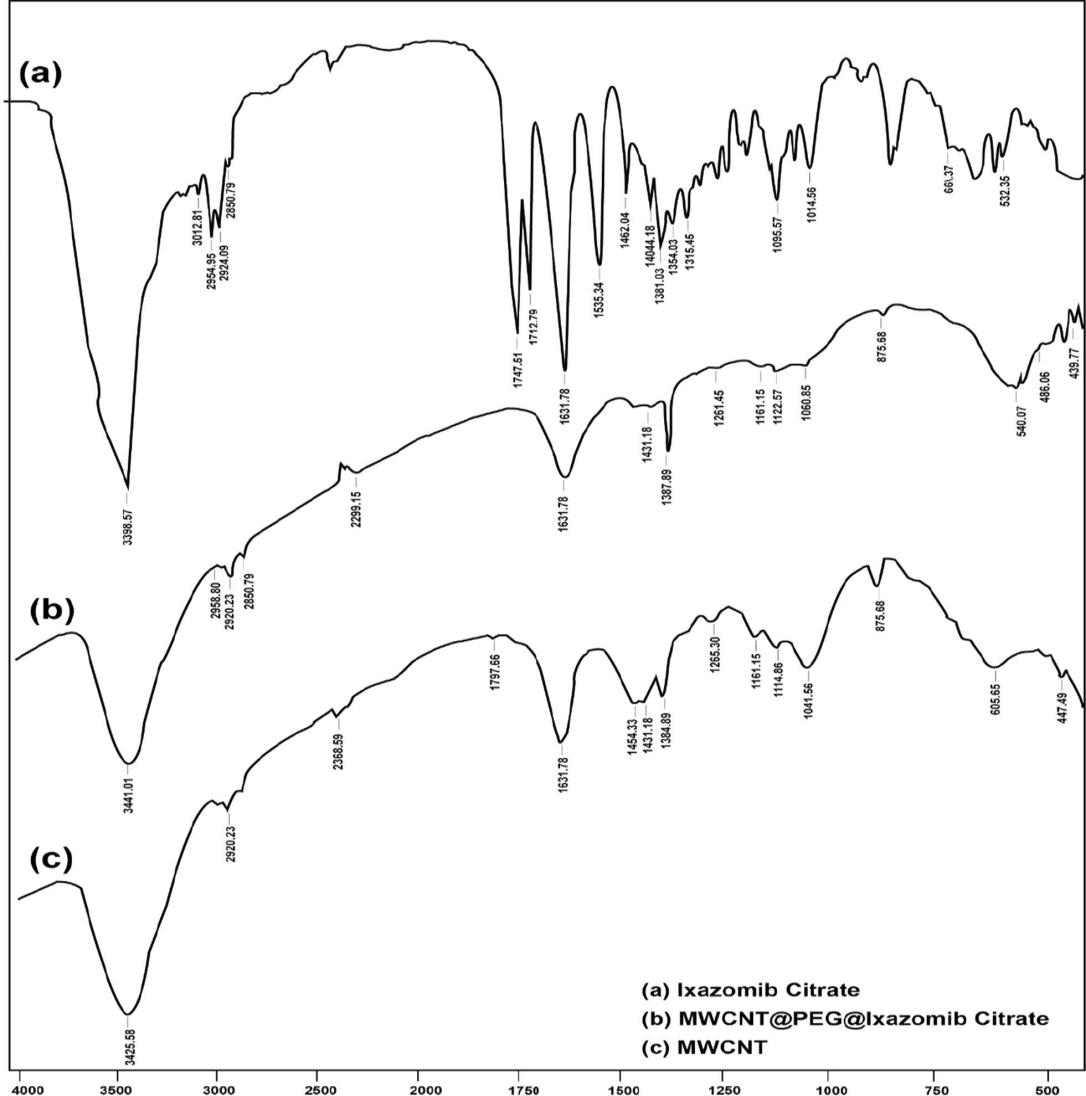
**a**–**c** FTIR of Ixazomib citrate, MWCNT@PEG@ Ixazomib citrate, and MWCNT, respectively

FTIR was used to determine the functional groups of Ixazomib, pristine, acidic and basic *o*MWCNTs, MWCNTs-PEG and MWCNTs-PEG-Ixazomib using a Shimadzu Fourier transform infrared spectrometer in the wavenumber range (400–4000 cm^−1^). The measurements were performed after mixing the material being studied with KBr in a ratio of 1:300 and proceeded at room temperature**.** FTIR analysis was used to confirm the loading of Ixazomib citrate to functionalized multi-walled carbon nanotubes (Fig. 2). In the case of the Spectrum of Ixazomib citrate [30], two bands appearing at 1747.51 and 1712.79 cm^−1^ that correspond to two O–H linked to two carboxylic groups in the drug have disappeared in the spectrum of MWCNTs@PEG@IXA, suggesting a covalent linkage between both O–H and COOH groups in the oxidized MWCNTs. In a comparison of the spectrum of Ixazomib Citrate before and after loading, bands at 1631.78 and 2850.79 cm^−1^ appeared at both spectra of Ixazomib Citrate before and after loading, while the spectrum of Ixazomib Citrate has bands at 1381.03, 2924.09, and 3398.57 cm^−1^ are shifted slightly to higher frequencies, 1384.89, 2958.8, and 3441.01 cm^−1^, respectively, after loading, indicating that confirm loading of Ixazomib Citrate to functionalized MWCNTs without decomposition. The spectra of MWNT and MWCNTs@PEG@IXA have the same bands at 1161.15, 1384.89 1431.18, and 1631.78 cm^−1^, that demonstrate that the presence of multi-walled carbon nanotubes in the MWCNTs@PEG@ IXA formula.

#### SEM

**Fig. 3:**
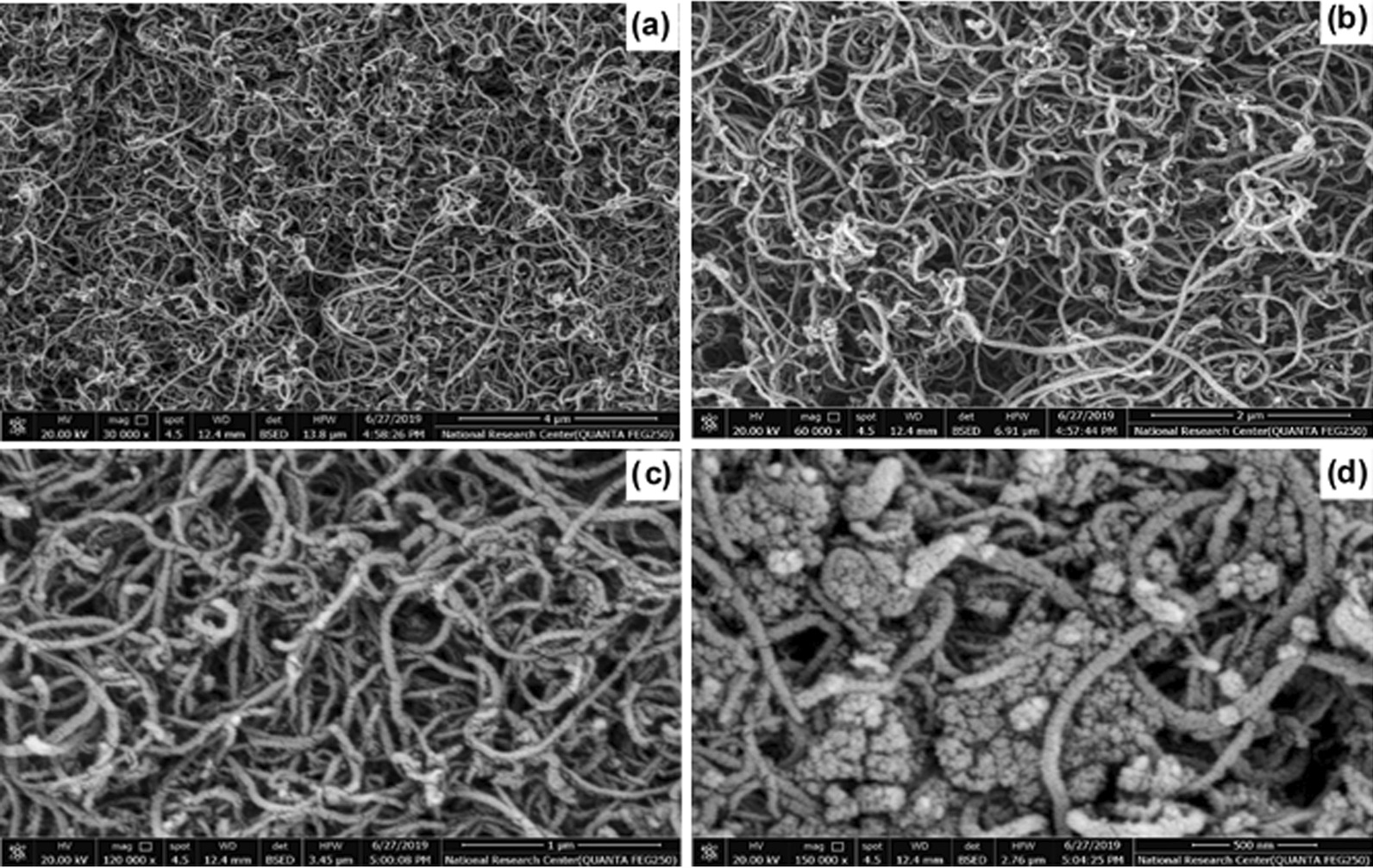
SEM images of **a**, **b** MWCNTs, **c** MWCNTs-COOH, **d** MWCNT-PEG-Ixazomib

First, the morphology, surface and subsurface of oMWCNT and MWCNTs-PEG-Ixazomib were examined using a scanning electron microscope (SEM). The diameter of the MWCNTs was determined to be < 50 nm and the MWCNTs-PEG composite was longer than 200 nm (Fig. 3a, b). The cross‑sectional width of MWCNTs was ~ 25 nm–52 nm and the pore diameter of MWCNTs was ~ 2–10 nm (Fig. 3c). Ixazomib was highly coated over MWCNTs-PEG in the MWCNTs-PEG-Ixazomib composite, and was investigated and confirmed by using SEM images that show different thicknesses in the range of 20–30 nm, 50 nm, and 70 nm for MWCNTs, functionalized MWCNTs-PEG and MWCNTs-PEG- Ixazomib, respectively, lower than the thickness of MWCNTs and functionalized MWCNTs-PEG were prepared in the study of Mohamed et al. [31]. Figure 3d shows that Ixazomib surrounded the raw MWCNTs substance to confirm that the raw Ixazomib was directly linked to the MWCNTs and Ixazomib had been coated over the surface of the MWCNTs. Decorating Ixazomib on the surface of MWCNTs, was confirmed because of disappearing the bulk of the MWCNTs surface as shown in (Fig. 3d). Furthermore, the size of the occupied space was observed to be not regular due to the random distribution of the drug.

#### TEM

**Fig. 4:**
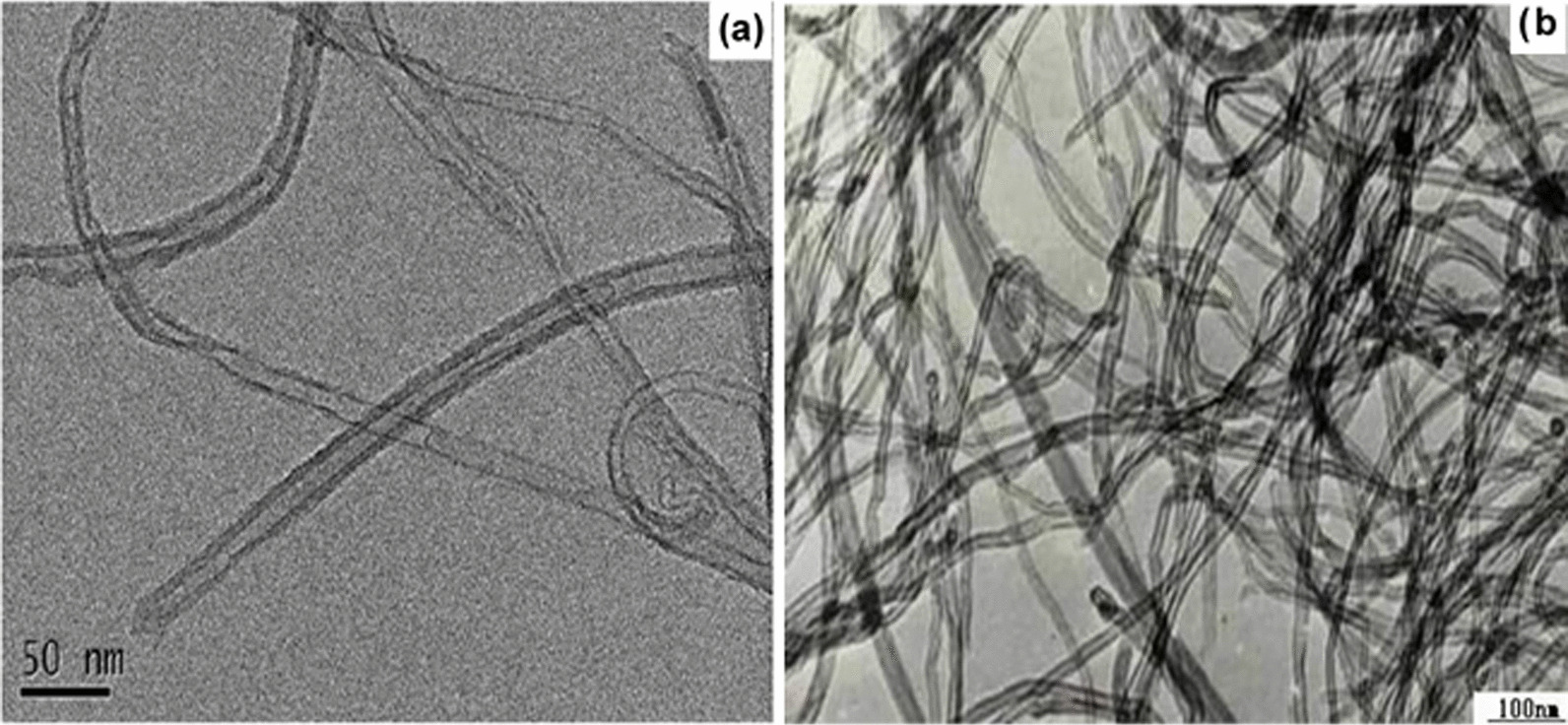
HRTEM images of MWCNTs **a** at 50 nm and **b** at 100 nm

**Fig. 5:**
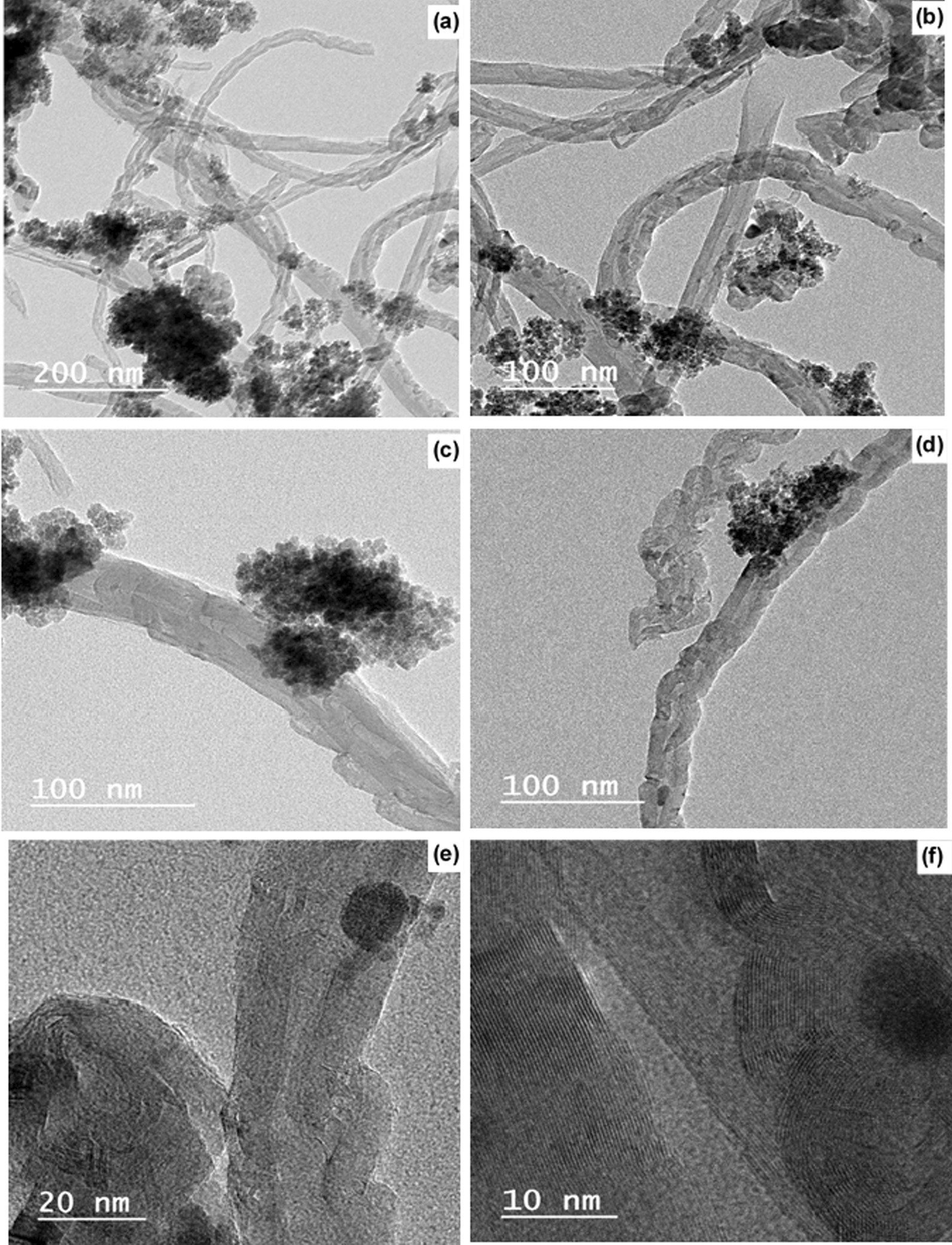
HRTEM images of MWCNTs-PEG-Ixazomib showing ixazomib citrate into and on the walls of CNTs

Using the transmission electron microscopy, the acidified MWCNTs and the MWCNTs-PEG-Ixazomib that were characterized as shown in (Fig. 4a, b) and (Fig. 5a, f) at different nanometers, were negatively stained with 1% H3PW12O40 for 1 min to get a higher magnification of TEM images. As shown in Fig. 5a, a smooth surface of acidified MWCNTs was obtained to apply for further applications.

Following the drug loading technique (Fig. 5c), the MWCNTs-PEG-Ixazomib had an uneven surface. The diameter of acidified MWCNTs was increased after loading Ixazomib to the surface of MWCNTs-COOH as shown in the results. MWCNTs-COOH and MWCNTs-PEG-Ixazomib were observed under TEM to identify morphological characterization. Their length ranged from a few hundred nanometers to a low magnification of 10 nm and MWCNTs-COOH and MWCNTs-PEG have diameter similar to similar materials that were performed in the study by Linlin et al. [32]. The drug Ixazomib citrate is well attached to the wall of carbon nanotubes.

#### TGA

**Fig. 6:**
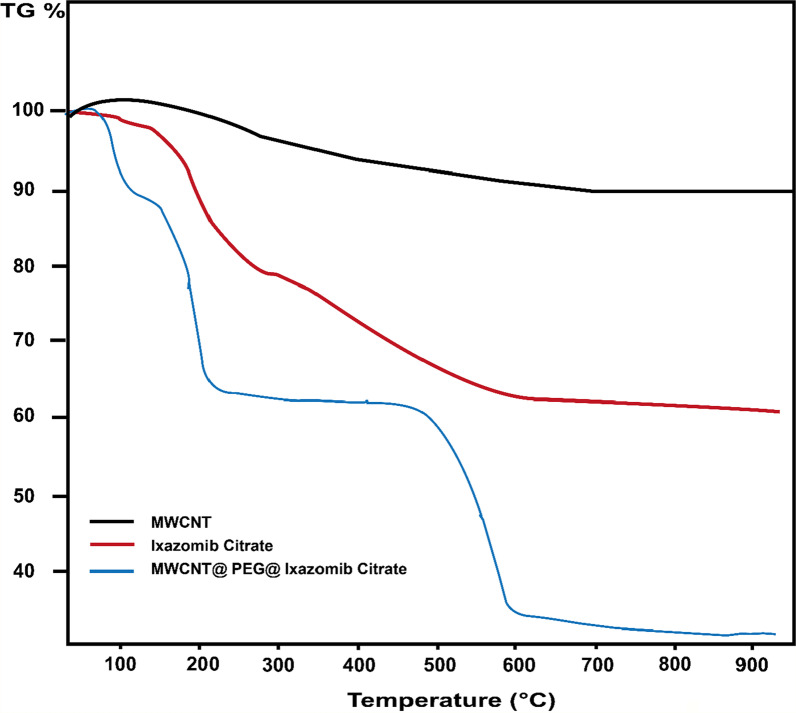
The TGA analysis for Ixazomib citrate, MWCNT, and the composite (MWCNT@PEG@Ixazomib

The TGA curve shows the thermal gravity analysis of Ixazomib citrate, MWCNTs, and the MWCNTs@PEG@Ixazomib composite (Fig. 6). The TGA result shows MWCNT weight increases first from 50 to 200 °C, because the sample may include unreacted graphite with heating of up to 200 °C, which led to the form of additional MWCNTs. When the temperature increased to 600, no weight loss for MWCNTs caused the functionalization of carbon nanotubes. While TGA was applied to characterize the thermal properties of the composite to confirm Ixazomib was incorporated within the composite, the composite lost less than 11.5% of its weight when it heated up to 150 °C. It indicates that 11.50 of the composites (after loading) consist of water and volatile solvents. Then, the temperature rose up to between the range (150–250 °C) and (450–600 °C), the composite losses were 25.5% and 25.9, respectively, which were attributed to the existence of Ixazomib citrate and PEG in the composite and linked to the functional surface of MWCNTs. 1% of the weights of composite loss in a temperature range between 250 and 450 °C, suggesting the low presence of inner gas incorporated in the composite. The compound has low impurities and inorganic compounds. It was shown from a loss of 0.6% of the composite weight when the temperature rose to higher than 600 °C.

#### DTG

**Fig. 7:**
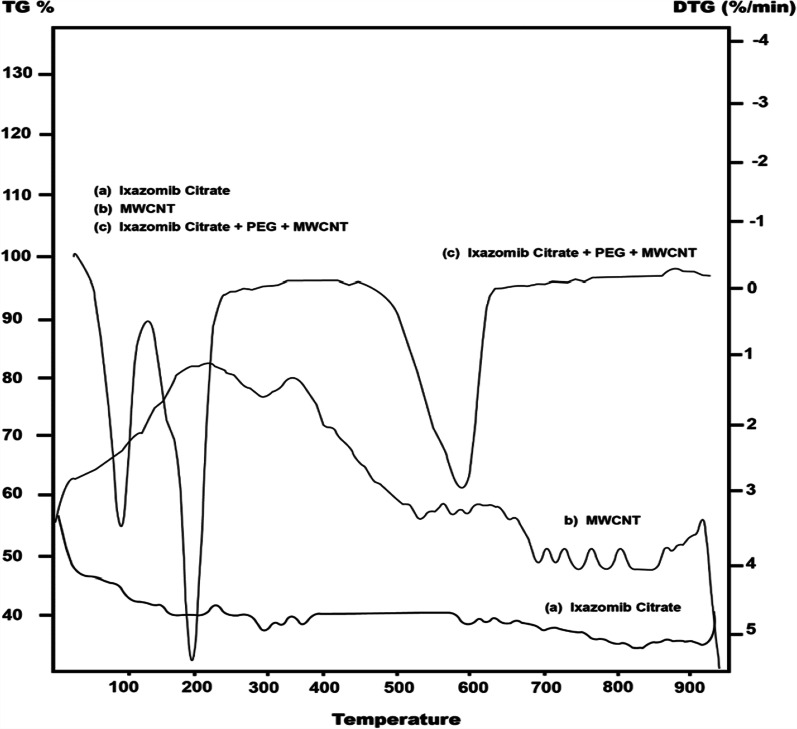
The DTG curve of Ixazomib citrate, MWCNT, and the composite (MWCNT@PEG@Ixazomib)

The DTG curve provides further evidence for covalent modification of MWCNTs (Fig. 7). A major peak at 278 °C could be attributed to the loss of the carbonyl groups bonded to MWCNTs. Alternatively, the DTG curve of MWCNTs PEGlated@ixazomib showed one peak at 538 °C, which can be assigned to the loss of MWCNTs. On the basis of previous results, the functionalization of MWCNTs-COOH and covalent modification of MWCNTs were successfully confirmed.

#### DSC

**Fig. 8:**
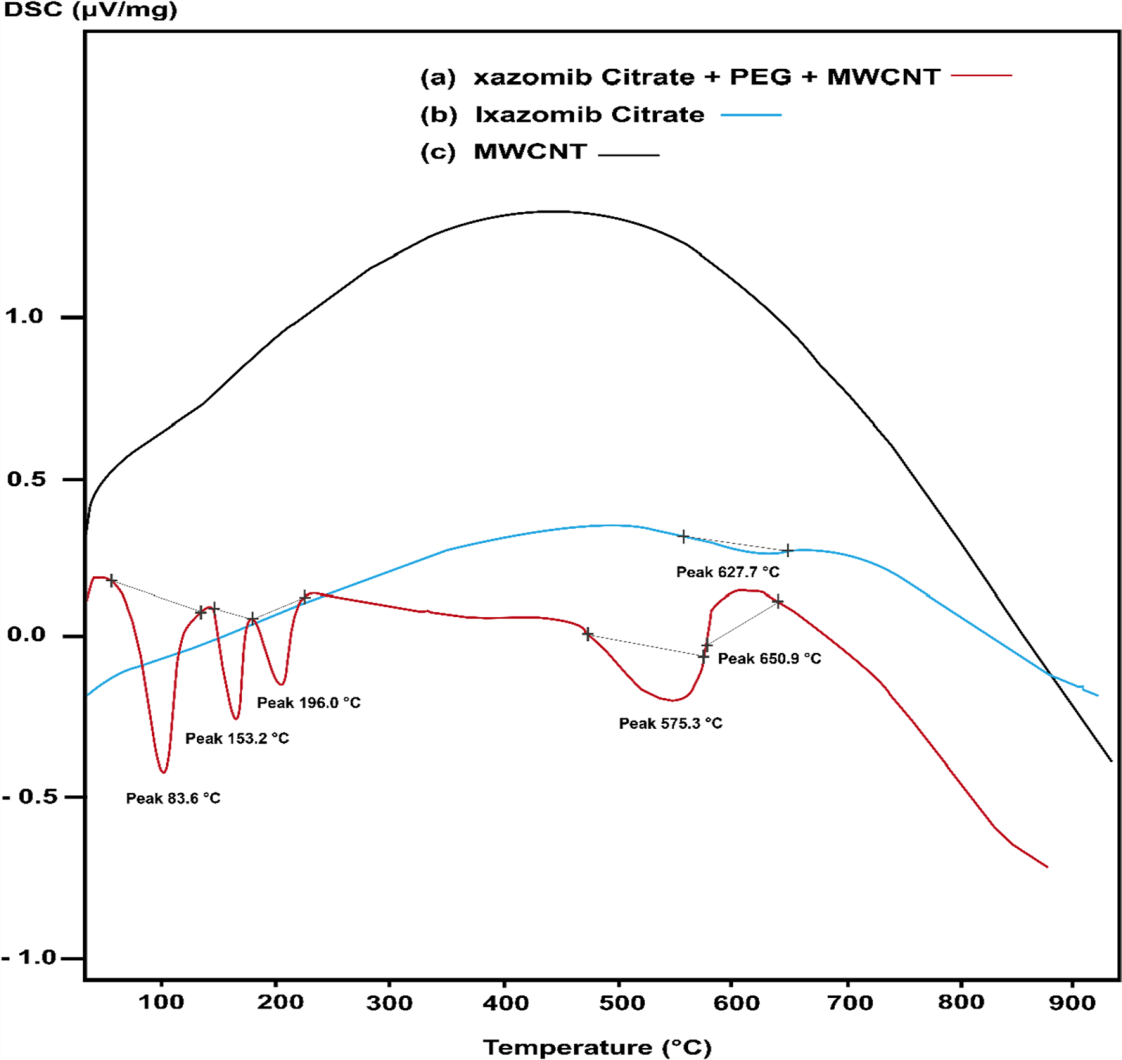
The DSC curve of Ixazomib citrate, MWCNT, and the composite (MWCNT@PEG@Ixazomib)

The DSC curve (Fig. 8) shows that the first peak at 83.6 °C is attributed to the transition temperature (TG) of the composite (MWCNTs@PEG@Ixazomib). It represents changes in the amorphous composite. The second clear endothermic peak is at 153.2 °C that is characteristic of mobility temperature of the composite. Multiple peaks at the DSC curve of the composite indicate that there are different morphologies in the composites (MWCNTs, drug (Ixazomib), polymers (PEG)). The exothermic peak at 650.9 °C on the DSC curve is evident in the decomposition of a composite. For this composite, the decomposition temperature changes to the highest temperature. While in Ixazomib citrate, a weak endothermic peak at 627.7 °C corresponds to the crystallinity of Ixazomib. In the case of MWCNTs, no characteristic peak was observed, suggesting the amorphous structure of multi-walled carbon nanotubes.

#### Ixazomib Citrate Loading by HPLC

Additional file 1: Fig. S4s shows the peak area of the unreacted Ixazomib citrate supernatant and the original Ixazomib citrate solution. The percentage of Ixazomib citrate loading capacity and entrapment efficiency were 44% and 96.13%, respectively, as calculated from Eqs. (1) and (2). Both the values are seen to be higher, compared to those obtained for PEGylated MWCNTs nanocomposite [33] thus, showing the significance of incorporation of MWCNT into the nanocomposite (Additional file 2).

#### Cell Culture and MTT Testing

**Fig. 9:**
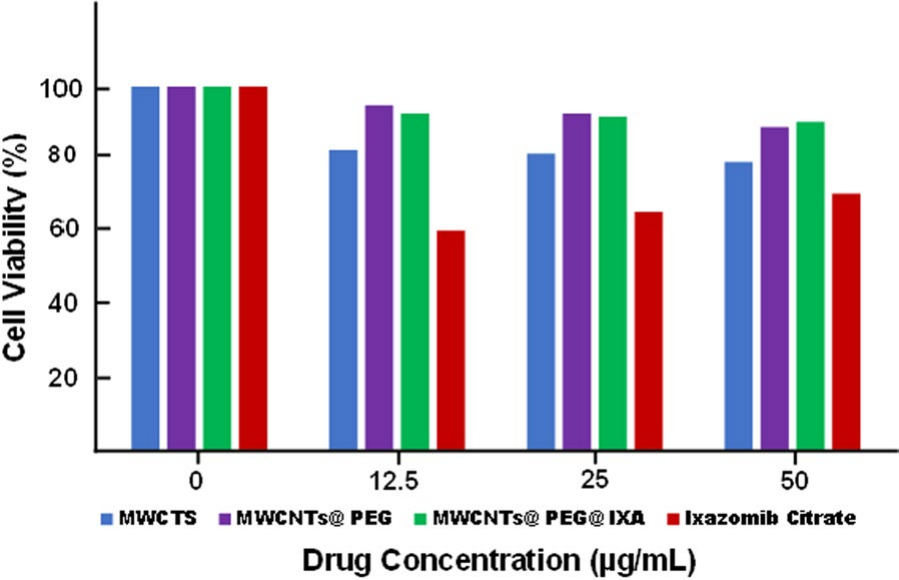
Percent cell viability of RPMI8226 cells after treated with free Ixazomib Citrate, MWCNTs@PEG@Ixa, MWCNTs@PEG, and MWCNTs for 48 h at 37 °C

The cell viability percentage of against RPMI8226 Human Multiple Myeloma Cancer Cells versus different concentrations of Ixazomib Citrate, MWCNTs, MWCNTs-PEG, MWCNTs-PEG-Ixazomib are displayed in Fig. 9. The cell viability of MWCNTs-PEG-Ixazomib in RPMI8226 cell lines was around 91% and higher than cell viability of MWCNTs and MWCNTs-PEG. Overall, both MWCNTs-PEG and MWCNTs-PEG-Ixazomib had no significant cytotoxicity even at high concentrations. The cytotoxicity of free Ixazomib citrate (69% cell viability of RPMI8226 cells) was higher than that of MWCNTs-PEG-Ixazomib (91% cell viability) at the same maximum concentration of Ixazomib citrate (50 µg/ml). The results of cytotoxicity suggested that Ixazomib citrate would still lead to cell death at high concentrations, and the cytotoxicity of MWCNTs could be overcome by functionalization with a biopolymer such as PEG. The cytotoxicity results would achieve long-term tumor inhibition of tumor growth with a minimum loss of drug and a minimum level of adverse effects.

## Conclusion

In summary, we have prepared a carrier by functionalization of MWCNTs by polyethylene glycol (PEG 600). This covalent functionalization creates active surfaces that can be used as a carrier for Ixazomib loading and release, which can be developed in a facile strategy. The characterization of MWCNTS- Ixazomib was performed using a simple preparation method. The characterization by FTIR, thermal and microscopic studies showed that Ixazomib molecules are loaded on the walls of MWCNTs-COOH using PEG 600 as a binder to give highly bounded MWCNTs-PEG- Ixazomib. This indicates that Ixazomib molecules and MWCNTs have been reacted to form covalent bonds in the presence of PEG 600 as a linker. The loading of Ixazomib has been studied by HPLC about (78% w/w). The cytotoxicity of MWCNTs-PEG-Ixazomib was evaluated in multiple myeloma cells using RPMI 8226 cell line to study MTT assay. It was shown that Ixazomib as a drug without loading had much greater cytotoxicity compared to MWCNTs-PEG-Ixazomib. This study indicates that the MWCNTs-PEG-Ixazomib composite is potentially useful for the delivery of therapeutic agents. In summary, to enhance the anticancer potency of Ixazomib, a novel ligand, these efforts can lead to the development of MWCNTs-PEG-Ixazomib as a clinical agent for the treatment of tumors such as multiple myeloma.

## Supplementary Information

**Additional file 1: Fig. S1s.** (a), (b) and (c) are TEM images of CNT bundles. (d) is an enlargement of region in (a) indicating a clear image of CNTs bundles at 100 nm. (e) and (f) are enlarged again in revealing that the CNTs are multi-walled. **Fig. S2s.** (a) (b). **Fig. S3s.** Figure 4. A diagram represents the process of drug loading to PEGylated MWCNTs. **Fig. S4s.** HPLC Chromatogram of Ixazomib Citrate Standard and Supernatant.

**Additional file 2.** Supplementary tables.

## Data Availability

The authors emphasize the availability of data and materials.

## Abbreviations

MWCNTs: Multi-walled carbon nanotubes
MLN2238: Ixazomib
MM: Multiple myeloma
PEG: Polyethylene glycol
DMF: Dimethyl formamide
DSC: Differential scanning calorimetry
TGA: Thermogravimetric analysis
DTG: Derivative thermal gravimetric analysis
FTIR: Fourier transform infrared spectroscopy
MWCNTs-PEG: Pegylated multi-walled carbon nanotube
SEM: Scanning electron microscopy
TEM: Transmission electron microscopy
HPLC: High-performance liquid chromatography
MTT: 3-(4,5-Dimethylthiazol-2-yl)-2,5-diphenyltetrazolium bromide
