# Supplementary material for: PEG@ Carbon Nanotubes Composite as an Effective Nanocarrier of Ixazomib for Myeloma Cancer Therapy
Source: Nanoscale Res Lett. 2022 Aug 5;17:72. doi: 10.1186/s11671-022-03707-2 (PMC9356125; doi:10.1186/s11671-022-03707-2)
Supplement: Supplementary file 1 — Additional file 1: Fig. S1s. (a), (b) and (c) are TEM images of CNT bundles. (d) is an enlargement of region in (a) indicating a clear image of CNTs bundles at 100 nm. (e) and (f) are enlarged again in revealing that the CNTs are multi-walled. Fig. S2s. (a) (b). Fig. S3s. Figure 4. A diagram represents the process of drug loading to PEGylated MWCNTs. Fig. S4s. HPLC Chromatogram of Ixazomib Citrate Standard and Supernatant. [file 11671_2022_3707_MOESM1_ESM.docx]

**Supporting Materials**

**PEG@ carbon nanotubes composite as a nanocarrier of Ixazomib for myeloma cancer therapy**

Hanady.A.Elgamal^1,2^, Samah Abdelsabour Mohamed^2^, Ahmed A.Farghali^1^, Abeer M. E. Hassan^3^,

^1^ Material Science and Nanotechnology Department, Faculty of Postgraduate Studies for Advanced Sciences (PSAS), Beni-Suef University, 62511 Beni-Suef, Egypt.

^2^ National organization of Drug Control and Research, Dokki, Egypt.

^3^ Analytical Chemistry Department, Faculty of Pharmacy, October 6 University, Egypt.

**Cell Culture and MTT testing:**

The cytotoxicity of Ixazomib Citrate and MWCNTs-PEG-Ixazomib on RPMI8226 human multiple myeloma cells were first evaluated with a MTT method. In the MTT assay, mitochondrial dehydrogenases cleave the tetrazolium ring and reduce MTT to insoluble dark blue formazan crystals. Only active mitochondria contain these enzymes and, therefore, the reaction only occurs in viable cells. Absorbance, directly proportional to cell viability, was determined at 450 nm in a BIOLINE ELIZA READER. The absorbance values were normalized by the controls and expressed as percentage viability. In our experiment, the cell viability of RPMI8226 has been decreased to a low percentage after treatment with 1–8 μM MWCNTs-PEG-Ixazomib in comparison to treatment with Ixazomib citrate only .

This result suggests that MWCNTs-PEG-Ixazomib has been interfered with the MTT assay in this study. We deduce the variation from different reports on the difference in MTT concentration used in the assay (the lower in MTT concentration, the less precipitation can be formed), time intervals between MTT addition and detection (the shorter in the time interval, the less interference be observed). Demonstration of MWCNTs-PEG-Ixazomib leading to significant cell growth inhibitory of RPMI8226 cells in a concentration-dependent manner was confirmed as shown in results

**Figures**

**Fig. 1s.** (a), (b) and (c) are TEM images of CNT bundles. (d) is an enlargement of region in (a) indicating a clear image of CNTs bundles at 100nm. (e)and (f) are enlarged again in revealing that the CNTs are multi-walled.

**Fig. 2s.** (a) (b)

**Fig. 3s.** Fig. 4. A diagram represents the process of drug loading to PEGylated MWCNTs.

**Fig. 4s.** HPLC Chromatogram of Ixazomib Citrate Standard and Supernatant.


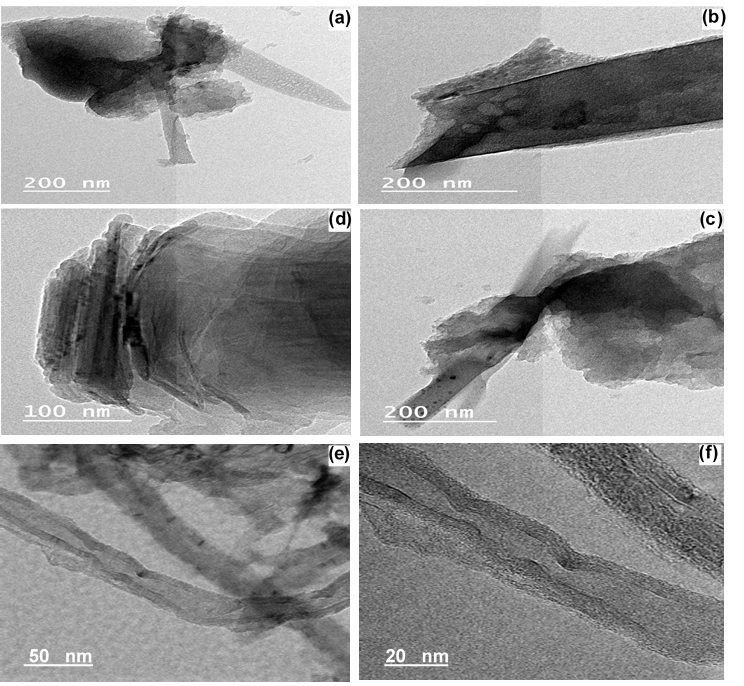


**Fig. 1s.**


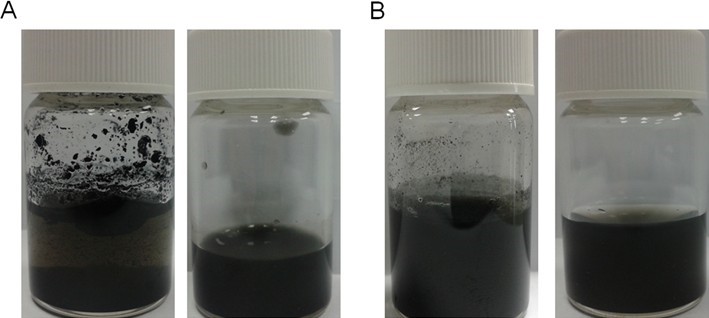


**Fig. 2s.**


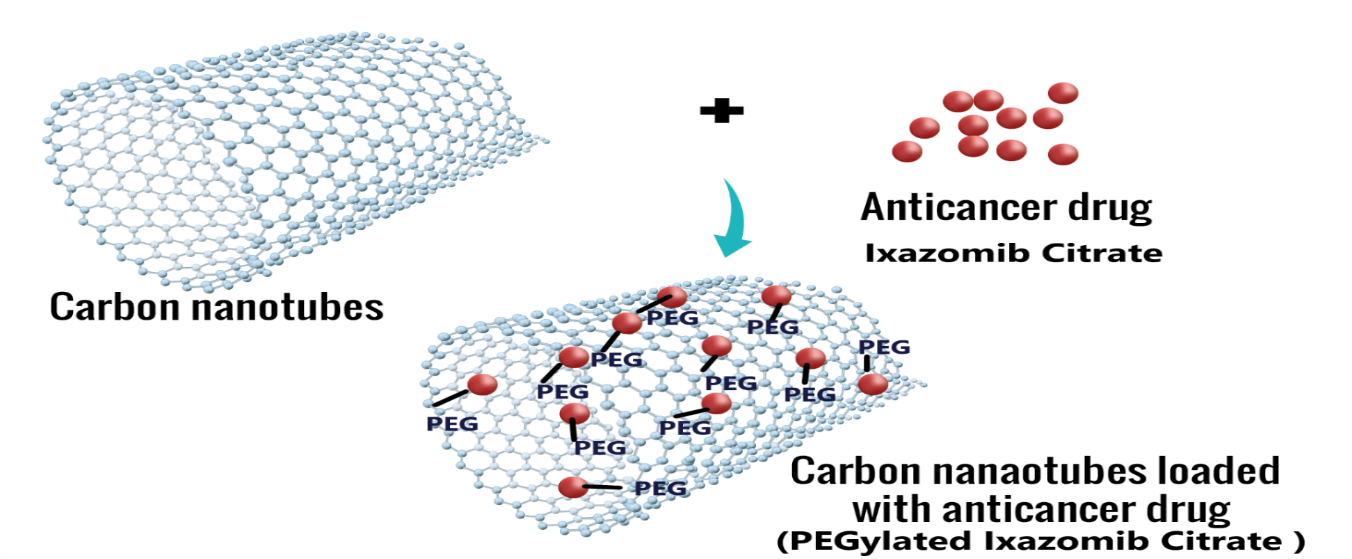


**Fig. 3s.**


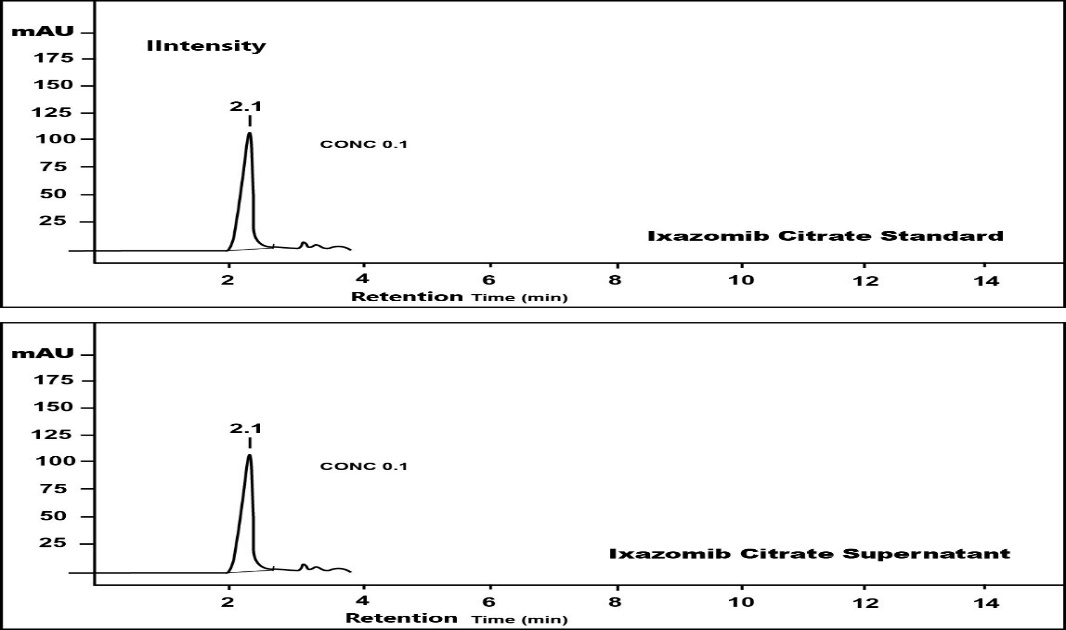


**Fig.** 4**s**.
