# Supplementary material for: PEG@ Carbon Nanotubes Composite as an Effective Nanocarrier of Ixazomib for Myeloma Cancer Therapy
Source: Nanoscale Res Lett. 2022 Aug 5;17:72. doi: 10.1186/s11671-022-03707-2 (PMC9356125; doi:10.1186/s11671-022-03707-2)

| **Ser** | **Sample** | **IC50 in RPMI8226 cell lines** |
| --- | --- | --- |
| 1 | Ixazomib Citrate | 17.63±0.58 |
| 2 | MWCNTs-PEG-Ixazomib | 9.30±0.3 |

| **Log Conc.** | **% Viability** **Ixazomib Citrate /RPMI8226** | **% Viability**  **MWCNTs-PEG-Ixazomib /RPMI8226** |
| --- | --- | --- |
| 2 | 39.2651 | 34.2939 |
| 1.39794 | 48.0548 | 42.4352 |
| 0.79588 | 56.9885 | 52.5216 |
| 0.19312 | 63.7608 | 61.5274 |
| -0.4089 | 73.1988 | 73.1268 |


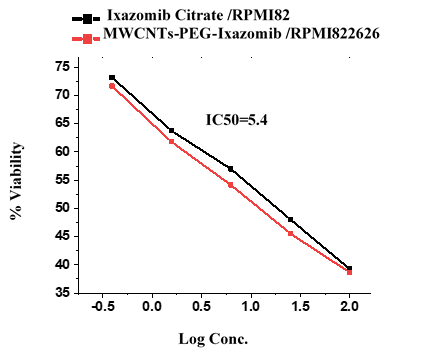

Supplement: Supplementary file 2 — Additional file 2. Supplementary tables. [file 11671_2022_3707_MOESM2_ESM.docx]
